# BNT162b2 mRNA Vaccination Leads to Long-Term Protection from COVID-19 Disease

**DOI:** 10.3390/vaccines9101164

**Published:** 2021-10-12

**Authors:** Claudia Rossi, Paola Lanuti, Ilaria Cicalini, Domenico De Bellis, Laura Pierdomenico, Piero Del Boccio, Mirco Zucchelli, Luca Natale, Bruna Sinjari, Giulia Catitti, Simone Vespa, Pasquale Simeone, Giuseppina Bologna, Ines Bucci, Katia Falasca, Jacopo Vecchiet, Liborio Stuppia, Vincenzo De Laurenzi, Damiana Pieragostino

**Affiliations:** 1 Center for Advanced Studies and Technology (CAST), “G. d’Annunzio” University of Chieti-Pescara, 66100 Chieti, Italy; claudia.rossi@unich.it (C.R.); paola.lanuti@unich.it (P.L.); ilaria.cicalini@unich.it (I.C.); domenico.debellis@studenti.unich.it (D.D.B.); laura.pierdomenico@unich.it (L.P.); piero.delboccio@unich.it (P.D.B.); m.zucchelli@unich.it (M.Z.); lucanatale1989@gmail.com (L.N.); catittig@gmail.com (G.C.); sv85@libero.it (S.V.); simeone.pasquale@gmail.com (P.S.); giuseppina.bologna@hotmail.it (G.B.); ines.bucci@unich.it (I.B.); stuppia@unich.it (L.S.); delaurenzi@unich.it (V.D.L.); 2 Department of Psychological, Health and Territory Sciences, School of Medicine and Health Sciences, “G. d’Annunzio” University of Chieti-Pescara, 66100 Chieti, Italy; 3 Department of Medicine and Aging Science, “G. d’Annunzio” University of Chieti-Pescara, 66100 Chieti, Italy; katia.falasca@unich.it (K.F.); jacopo.vecchiet@unich.it (J.V.); 4 Department of Innovative Technologies in Medicine and Dentistry, “G. d’Annunzio” University of Chieti-Pescara, 66100 Chieti, Italy; b.sinjari@unich.it; 5 Department of Pharmacy, “G. d’Annunzio” University of Chieti-Pescara, 66100 Chieti, Italy; 6 Clinic of Infectious Diseases, S.S. Annunziata Hospital, 66100 Chieti, Italy

**Keywords:** BNT162b2, SARS-CoV-2, vaccines, anti-S1 IgG, spike-specific T-cells

## Abstract

The efficacy of SARS-CoV-2 mRNA-based vaccines in preventing COVID-19 disease has been extensively demonstrated; however, it is of uttermost importance to acquire knowledge on the persistence of immune-protection both in terms of levels of neutralizing antibodies and specialized memory cells. This can provide important scientific basis for decisions on the need of additional vaccine doses and on when these should be administered thus resulting in an improvement in vaccination schedules. Here, we briefly report the changes in antibody levels and cellular immunity following BNT162b2 administration. We show an important fall in anti S1-Spike antibodies in BNT162b2 vaccinated subjects overtime, paralleled by a contextual consolidation of specific spike (S) T-cells, mainly of the CD8+ compartment. Contrariwise, CD4+ S-specific response shows a considerable interindividual variability. These data suggest that the well-known antibody drop in vaccinated subjects is replaced by memory cell consolidation that can protect from severe adverse effects of SARS-CoV-2 infection.

## 1. Introduction

The efficacy of SARS-CoV-2 mRNA-based vaccines in terms of reduction of infection spreading and more importantly in terms of reduction of disease severity, hospitalizations, and death has been widely demonstrated [1,2,3,4,5,6].

However, the duration of the immune protection following vaccination is still not well defined. The evaluation of vaccine efficacy is challenging, particularly in terms of duration of immune responses [7]. Numerous authors have pointed out the importance of immunological profile, consisting of memory B cells, antibodies, memory CD4+ T-cells, and memory CD8+ T-cells, for durable protective immunity [8]. Thus, it is becoming evident that the knowledge of immune memory may be crucial for a better evaluation of the duration of immune response to SARS-CoV-2 vaccination. Previous studies have shown that the SARS-CoV-2 (BNT162b2) booster vaccination produces high levels of neutralizing antibodies which, as expected, decrease over time [6,7,9,10,11], while it has been demonstrated that T-cell immunity has a key role for a durable immune memory response as protection against SARS-CoV-2 infection, and memory T-cell responses can persist for many years [12,13,14]. Of interest, the magnitude of ORF1ab-specific SARS-CoV-2 T-cell responses during infection of adults associates with reduced duration of illness but not with symptom severity [12]. Moreover, it has been shown that CD8+ T-cell responses increase in time post SARS-CoV-2 vaccines [12,13,15]. Therefore, taking into consideration that antibodies and memory T-cells, both CD4+ and CD8+, are all involved in protective immunity for both humoral and cell-mediated immune responses against COVID-19, we assessed the longitudinal stability of SARS-CoV-2 T responses in BNT162b2 vaccinated adults as well as the changes in anti-SARS-CoV-2 IgGs, 15 and 150 days following the boost with the second dose of vaccine [11].

## 2. Materials and Methods

### 2.1. Patients

Samples were collected at the Center for Advanced Studies and Technology (CAST), “G. d’Annunzio” University of Chieti-Pescara, where there is the “Newborn screening laboratory” for dried blood spot (DBS) and blood collection. A total of 60 subjects were enrolled for IgG determination, 20 CTRL (age 45.95 ± 19.13) and 40 (age 36.57 ± 10.32) BNT162b2 vaccinated subjects (analyzed 15 and 150 days after the second dose administration). A homogeneous group of participant was enrolled taking into consideration that age deeply influences the IgG titer, as already reported [16,17]. Forty-two independent subjects were studied for memory T-cell expression. Five subjects were analyzed twice (within 2 two months and after two months from BNT162b2 second dose inoculation). No concomitant pathologies were declared by the enrolled donors. Data from recruited donors are reported in Table S1.

### 2.2. Anti-S1 Spike IgG Measurement

IgG antibodies to SARS-CoV-2 were measured as already described [11] by a fully automated solid phase DELFIA (time-resolved fluorescence) immunoassay in a few drops of blood collected and dried on filter paper through the use of GSP^®^/DELFIA^®^ Anti-SARS-CoV-2 IgG kit time-resolved fluoroimmunoassay on a GSP instrument (PerkinElmer^®^, Turku, Finland. IgG levels were calculated as ratio of fluorescence of the sample over the calibrator. The test screened as positive subjects having IgG levels above the laboratory 1.2 cut-off.

### 2.3. Peripheral Blood Mononuclear Cells (PBMC) Isolation, Stimulation and Staining for Flow Cytometry Analyses

PBMC were isolated using Ficoll–Paque (Pharmacia, Uppsala, Sweden) gradient centrifugation from citrated peripheral blood samples. PBMC were then resuspended in RPMI-1640 medium (Roswell Park Memorial Institute) from Bio-Whittaker, Verviers, Belgium, containing 10 mM of L-glutamine (Biochrom, Berlin, Germany), 0.1 mg/mL of penicillin and streptomycin (Biochrom), and 10% fetal bovine serum (Biochrom) [18]. The cell concentration was adjusted to 2 × 10^6^ cells/mL. PBMC were stimulated with a pool of spike peptides (PepTivator S, cat. 130-126-701, PepTivator S1, cat. 130-127-048, PepTivator S+, cat. 130-127-312, Miltenyi Biotec, Bergisch Gladbach, Germany) at the recommended concentrations for 16 h (37 °C, 5% of CO_2_), while negative controls were treated with the same amount of vehicle used to dissolve the peptide mix [14,15,19]. After 2 h of stimulation, samples were treated with 6.5 µL of GolgiStop (554724, BD Biosciences, La Jolla, CA, USA). The staining was carried out as already published [11], using the reagent list reported in Table S2. Samples were finally acquired by flow cytometry (CytoFLEX, Beckman Coulter, Chaska, MN, USA).

### 2.4. Flow Cytometry Analyses

TCR-dependent activation induced marker (AIM) assay [11,20] and flow cytometry with intracellular cytokine staining assays (ICS) were carried out as reported [11,15,20]. A representative example of gating strategy used for all analyses is depicted in Figure S1. Instrument performances and data reproducibility were sustained and checked by using the beads CytoFLEX Daily QC Fluorospheres (ref. B53230, Beckman Coulter). To assess non-specific fluorescence, fluorescence minus one (FMO) controls were used. Compensation was calculated using VersaComp Antibody Capture Beads (ref B22804, Beckman Coulter) and single stained samples. Data were analyzed using FlowJo v 10.7.2 (BD Biosciences) software. Functional subsets were obtained by boolean gating. Frequencies of T-cell responses were displayed as percentages of CD4+ or CD8+ T-cells. T-cells producing at least 1 of the tested cytokines in the CD4+ and CD8+ T-cell compartments were considered specific for S protein stimulation.

### 2.5. Statistics

All statistical tests were performed using GraphPad Prism 9 (GraphPad Software, San Diego, CA USA), XLSTAT2021 (Addinsoft, New York, NY, USA). A D’Agostino–Pearson normality test was applied and the Kruskal–Wallis test and Dunn’s multiple comparisons test were used to assess the statistical differences.

## 3. Results and Discussion

**Figure 1:**
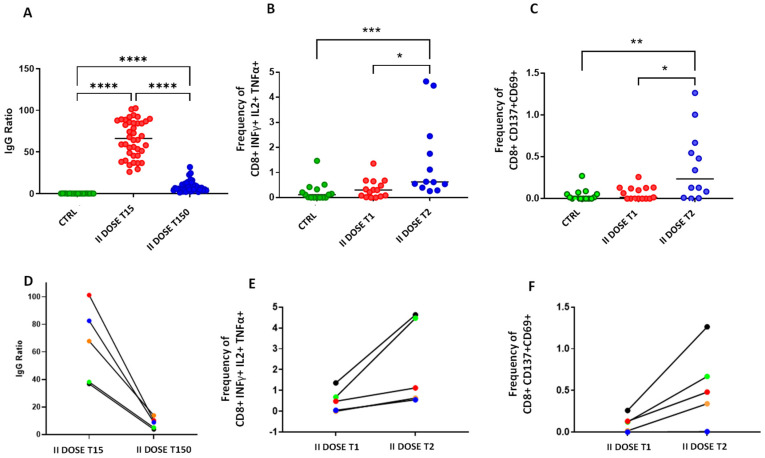
Panel (**A**): Levels of anti-SARS-CoV-2 IgG measured in: A control group that was not infected or vaccinated (CTRL, green dots); in a group of vaccinated subjects 15 and 150 days after the second dose of BNT162b2 (respectively: red dots, II DOSE T15 and blue dots II DOSE T150). Panels (**B**,**C**): Frequencies of S-specific CD8+ cells producing cytokines (INFγ+ IL2+ TNFα+ in Panel (**B**) and CD137+CD69+ in Panel (**C**)) are shown in SARS-CoV-2 unexposed healthy donors who never received any anti-SARS-CoV-2 vaccine (CTRL), in vaccinated donors analyzed between 0 and 2 months after the second dose (red, II DOSE T1), and between 3 and 5 months after the second dose (blue II DOSE T2). Individual data points are represented as scatter dot plots with lines showing the median value. Panel (**D**): IgG anti-SARS-CoV-2 levels measured in five vaccinated donors after 15 and 150 days after the second dose of BNT162b2 vaccine. Panel (**E**): Frequency trend of S-specific CD8+ T-cells INFγ, IL2, and TNFα positive in the five donors vaccinated subjected: thirthy days after the seconds dose (II DOSE T1) and approximately 100 days after the second dose (II DOSE T2). Panel (**F**): Frequency trend of S-specific CD8+, CD137+, and CD69+ T-cells. * indicates *p* value < 0.05, ** indicates *p* value < 0.01, *** indicates *p* value < 0.001, **** indicates *p* value < 0.0001.

We compared the IgG levels (Figure 1, Panel A) in control (green dots) and in BNT162b2 vaccinated subjects (red and blue dots). In particular, we reported the IgG ratio at 15 days after the second dose (red dots), which represented the maximum IgG ratio [11], showing a mean ratio of 66.23 ± 22.10, and after 150 days from the second dose administration (blue dots), showing a mean ratio of 8.82 ± 6.58. We observed a rapid increase of IgG levels following administration of the second dose as compared to CTRL (*p* < 0.0001), but also a dramatic fall after 150 days (*p* < 0.0001) to Dunn’s multiple comparisons post-test. However, levels of IgG remained positive in 100% of the tested individuals and were significantly higher than controls after 150 days from the second dose (*p* < 0.0001).

We then evaluated SARS-CoV-2-specific T-cell response in non-vaccinated controls and in BNT162b2 vaccinated donors at two different time points: between 0 and 2 months (II DOSE T1) and between 3 and 5 months (II DOSE T2) following administration of the second dose. Direct ex vivo CD4+ and CD8+ T-cell responses were evaluated analyzing key antiviral cytokine production in S-specific reactive T-cells as a read-out (Figure S1). We showed that the frequency of CD8+ INFγ+ IL2+ TNFα+ as well as CD8+ CD137+CD69+ significantly increases later after the second dose administration. Actually, as reported in Figure 1, Panels B and C, the frequencies of CD8+ INFγ+ IL2+ TNFα+ as well as CD8+ CD137+CD69+ are significantly higher after the second dose administration in II DOSE T2 (3–5 months) compared to II DOSE T1 (0–2 months). Table S3 of Supplementary Materials summarizes the mean of each measured biomarkers.

While the levels of antibodies and the T-cell response were analyzed in different groups of donors, we were able to perform both analyses at different time points in 5 of them (Figure 1, Panels D–F).

Our results showed a significant increase of S-specific CD8+ T-cells responses over time (Panels E and F) in contrast to the dramatic decrease of the antibody titers (Panel D), plots for one representative subject are shown in Figure S2.

To confirm the appropriate use of cytokine production as a surrogate measure of spike-specific T-cell responses, the expression of activation-induced markers (AIM assay) were also analyzed. AIM assay also showed a higher response in BNT162b2-vaccinated donors compared to non-vaccinated adults. No significant differences were found in CD4+ T-cells when the two time points were compared (Figure S3) and no correlation was found between the immunological response and age due to the fact that the study was conducted on a homogenous age group (Table S4).

Our results clearly indicate that the antibody titre decreases rapidly after vaccine of over than 80% in respect the maximum titre, on the contrary there is an increase in the frequencies of memory T-cells. These results are particularly encouraging considering that in animal models of reinfection, spike-specific CD8+ T-cell responses were able to compensate for inadequate antibody production, also providing an immune correlate of protection [21]. In fact, it is known that memory CD8+ T-cells are antigen-specific and long-lived T-cells, and they provide an enhanced protective response when the same antigen is encountered again [22]. Memory CD8 T-cells persist, populate peripheral organs, and, upon the specific antigen re-encounter, they immediately proliferate vigorously, execute cytotoxic functions, and secrete effector cytokines [23]. For these reasons, the severity of COVID-19 has been shown to be reduced by rapid and early recruitment of established immune response [24,25], suggesting that the presence of spike-specific CD8+ T-cells following vaccination predicts a paucisymptomatic disease. Moreover, as it has been recently demonstrated [26], SARS-CoV-2-specific T-cells can be detected in the absence of antibodies in patients with previous COVID-19 disease. These data, combined with our results suggest that T-cell responses would be more sensitive indicators of SARS-Co-V-2 exposure than antibodies. It would therefore be useful to have a tool to easily evaluate T-cell response upon vaccination; however, at present no simple, fast, or inexpensive method is available. In addition to the flow cytometric approach used in this manuscript, an alternative method for the evaluation of specific T-cell activation is the ex vivo enzyme-linked immunospot (ELISPOT) assay that however is as laborious and time consuming but less accurate [27,28]. Further, a tool to easily evaluate T-cell response could be important in cases where exposure to SARS-CoV-2 induce virus-specific T-cell responses without seroconversion [26].

We believe that this study underlies the advantage of DBS sampling in assessing antibody response in large populations being simple reproducible and less expensive than most other methods. While studies with larger number of donors of different age groups should be performed to confirm our studies, the data presented suggest that in terms of disease severity, the administration of a third dose of vaccine can be postponed as far as memory T-cells can be detected, while protection from infection, mostly due to antibody levels, requires a new booster dose already after 5 months. Clearly, this has important implications for public health, indeed indicating that protection from severe consequences is long lasting, potentially even for years, thus supporting the hope that it will not be necessary to continue administration of vaccine doses if and when the virus enters an endemic phase.

## Data Availability

Data are contained within the article and Supplementary Materials.

## Supplementary Materials

The following are available online at https://www.mdpi.com/article/10.3390/vaccines9101164/s1. Figure S1: Gating strategy for SARS-CoV-2 S-reactive T-cell identification after the first dose of vaccines, Table S1: Clinical details of enrolled patients, Table S2: Reagent list for flow cytometry analyses, Figure S2: Details of analysis for one vaccinated subjects 15 days and five months after the second dose inoculation, Figure S3: CD4+ memory T-cell frequencies analysed at two different time points: within (T1) and after (T2) two months from the second dose BNT162b2 administration, Table S3: Mean of measured biomarkers, Table S4: Correlation analysis between age of the subjects and their immunological responses in terms of antibody levels and CD8+ cells frequencies.
