# Supplementary material for: BNT162b2 mRNA Vaccination Leads to Long-Term Protection from COVID-19 Disease
Source: Vaccines (Basel). 2021 Oct 12;9(10):1164. doi: 10.3390/vaccines9101164 (PMC8538967; doi:10.3390/vaccines9101164)
Supplement: Supplementary file 1 [file vaccines-09-01164-s001.zip › vaccines-1394774-supplementary.pdf]

vaccines

## Supplementary Material

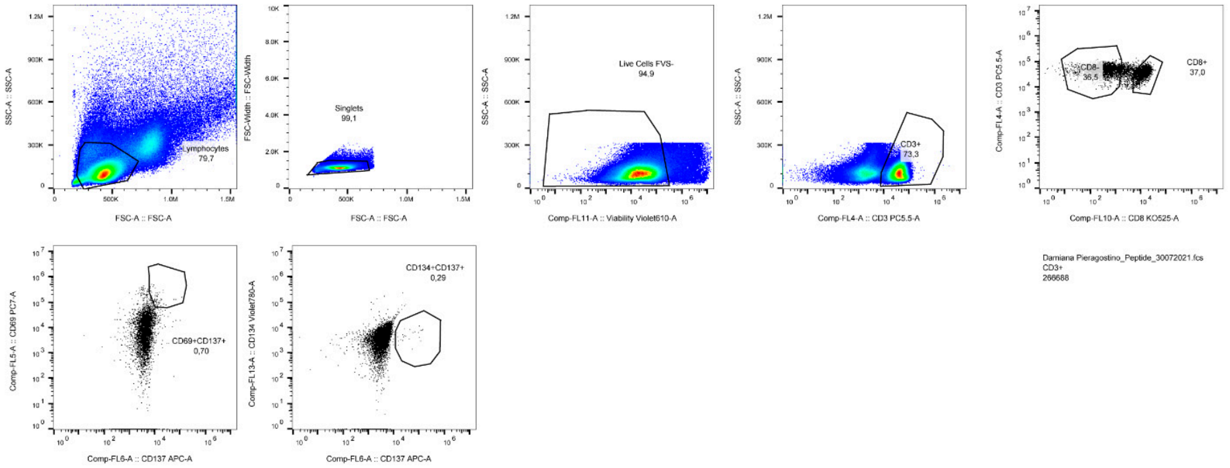


**Figure S1. Gating strategy for SARS-CoV-2 S-reactive T cell identification.** Lymphocytes were first selected in a forward scatter (FSC) area versus side scatter (SSCA) area dot-plot. Aggregates were next excluded (FSC-A/FSC-width [W] plot) and live cells were identified. CD3+ T-cells were then gated (CD3/SSCA dot-plot). T-cells were split into CD8- (CD4+ cells) and CD8+ subtypes (CD8/CD3). TCR-dependent activation induced markers (AIM) from a representative donor are also represented for CD8+ T cells (CD137/CD69 dot-plot) and CD4+ T cells (CD137/CD134 dot-plot).

**Table S1: Clinical Details of enrolled patients**

| Subjecs | | Number of subjects for IgG | Number of Subjects for Memory | | Follow up | Age of IgG levels | Age of Follow-Up subjects |
|---|---|---|---|---|---|---|---|
| **CTR** | | 20 | 15 | | | 45,95 ± 19,13 | |
| **T1** | | 40 | 15 | | 5 | 36,57 ± 10,32 | 42,2 ± 5,54 |
| **T2** | | 40 | 12 | | 5 | 36,57 ± 10,32 | 42,2 ± 5,54 |

**Table S2:** Reagent List for Flow Cytometry Analyses

| Specifity | Clone | Fluorochrome | Amount *per* test | Type staining | Catalogue Number |
|---|---|---|---|---|---|
| CD197 | 2-L1-A | PE | 5 µl | Surface | 566742 |
| CD3 | SK7 | PerCP-Cy5.5 | 20 µl | Surface | 332771 |
| CD69 | FN50 | PE-Cy7 | 5 µl | Surface | 561928 |
| CD45RA | HI100 | APC-H7 | 5 µl | Surface | 560674 |
| CD8 | SK1 | V500-C | 5 µl | Surface | 647457 |

| | | | | | |
|---|---|---|---|---|---|
| FVS 575V | - | - | 1 µl | Surface | 565694 |
| CD134 | L106 | BV768 | 5 µl | Intracellular | 744746 |
| CD137 | 4B4-1 | APC | 20 µl | Intracellular | 550890 |
| IFNg | B27 | FITC | 20 µl | Intracellular | 552887 |
| TNFa | MAb11 | Alexa Fluor™700 | 5 µl | Intracellular | 557996 |
| IL-2 | 5344.111 | BV711 | 5 µl | Intracellular | 563946 |
| R-phycoerythrin (PE); peridinin chlorophyll protein-cyanine 5.5 (PerCP-Cy 5.5); PE-Cyanine 7 (Cy7); Allophycocyanin-Hilite®7 (APC-H7); Fixable Viability Stain (FVS); Brilliant Violet (BV); Allophycocyanin (APC); Fluorescein isothiocyanate (FITC). All the reagents listed in the table are from BD Bdiosciences. | | | | | |

**Figure S2:** Spike specific cytokine production within (T1) and after (T2) two months from the second dose BNT162b2 administration. Cytokines (interferon [IFN]-$\gamma$, CD40L, tumour necrosis factor [TNF]-$\alpha$ and interleukin [IL]-2) were individually analysed for each subset (CD4+ and CD8+), in a representative BNT162b2 vaccinated donor at two different time points after the second dose administration, using the corresponding DMSO control to assess and subtract the background.

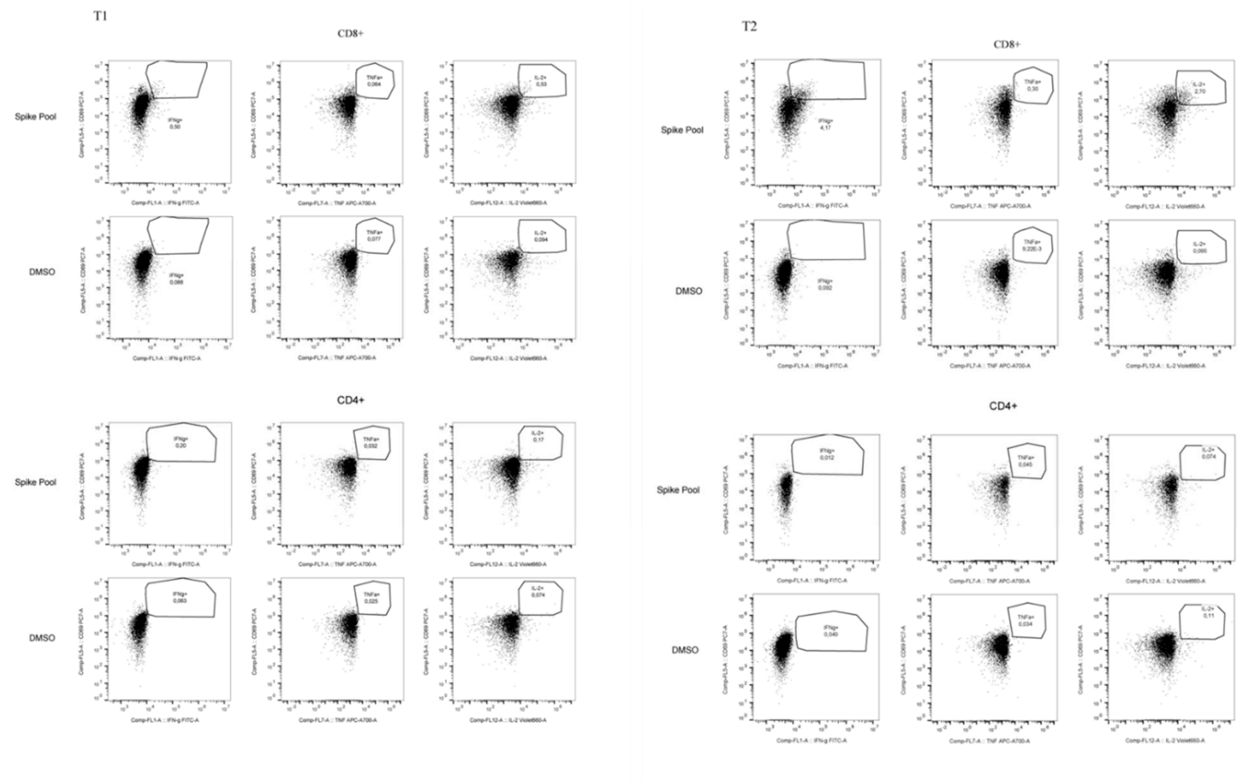

**Figure S3:** CD4+ memory T-cell frequencies analysed at two different time points: within (T1) and after (T2) two months from the second dose BNT162b2 administration.

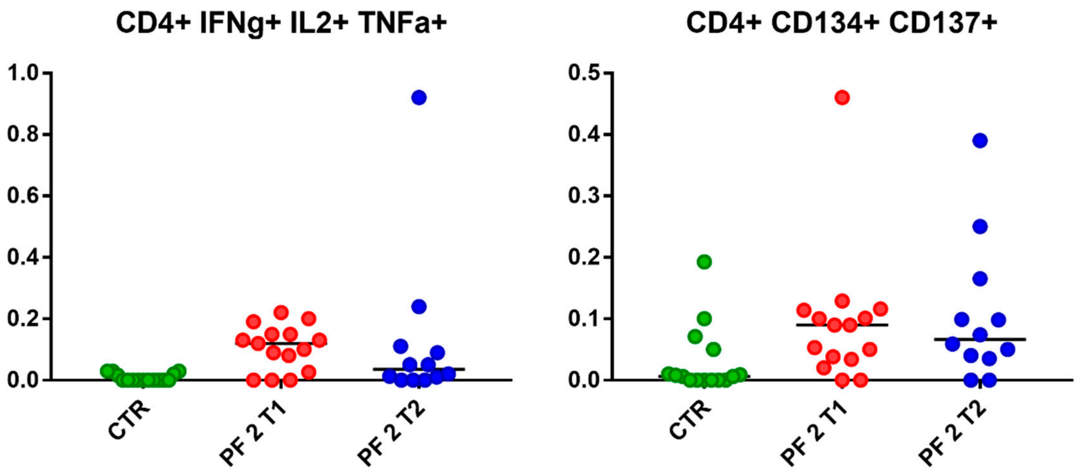


**Table S3 Mean of measured biomarkers**

| Table S3 | Levels of IgG | Age of Memory subjects | Frequencies CD8+IFNg+ IL2+ TNFa+ | Frequencies CD8+CD137+CD69+ | Frequencies CD8+IFNg+ IL2+ TNFa+ Follow-Up subjects | Frequencies CD8+CD137+CD69+ |
|---|---|---|---|---|---|---|
| **CTR** | 0,2185 ± 0,0631 | 46,07 ± 11,73 | 0,224 ± 0,384 | 0,038294 ± 0,071532 | | |
| **VACC T1** | 66,23375 ± 22,10569 | 44,13 ± 9,3 | 0,3497 ± 0,373 | 0,0636 ± 0,07877 | 0,5132 ± 0,5540 | 0,1054 ± 0,1044 |
| **VACC T2** | 8,819 ± 6,58186 | 40,75 ± 6,94 | 1,47625 ± 1,5757 | 0,38883 ± 0,41877 | 2,2816 ± 2,0848 | 0,552 ± 0,4663 |

**Table S4: Correlation analysis between age of the subjects and their immunological responses in terms of antibody levels and CD8+ cells frequencies**

| | Age | Age | Age |
|---|---|---|---|
| | vs. | vs. | vs. |
| | CD8+IFNg+ IL2+ TNFa+ | CD8+CD137+CD69+ | IgG at time of collection |
| 95% confidence interval | -0,4763 to 0,195 | -0,5475 to 0,1006 | -0,3996 to 0,3325 |
| R squared | 0,02526 | 0,06321 | 0,001502 |
| | | | |
| P value | | | |
| P (two-tailed) | 0,3770 | 0,1581 | 0,8418 |
| P value summary | ns | ns | ns |
